# South-South cooperation as a mechanism to strengthen public health services in Africa: experiences, challenges and a call for concerted action

**DOI:** 10.11604/pamj.2017.28.40.12201

**Published:** 2017-09-15

**Authors:** Olushayo Olu, Amos Petu, Martin Ovberedjo, Diane Muhongerwa

**Affiliations:** 1 WHO Country Office, Kigali, Rwanda; 2 WHO Intercountry Support Team for Eastern and Southern Africa, Harare, Zimbabwe; 3 WHO Country Office, Gaborone, Botswana

**Keywords:** Horizontal development cooperation, South-south cooperation, effective public health services delivery, health system strengthening, aid effectiveness, sustainable development goals, Africa

## Abstract

Implementation of new models of development cooperation have been on the increase lately. Coupled with this are calls for use of horizontal development cooperation mechanisms such as South-South Cooperation (SSC) as a way to enhance aid effectiveness in the health sector of developing countries. In this case series, we review recent experiences in the application of SSC initiatives to two public health situations in Africa to demonstrate the veracity of this new paradigm. Our review highlight the immense benefits associated with the use of SSC for health and provide evidence for increasing use of horizontal development coordination mechanisms to strengthen public health services delivery and socioeconomic development among African countries. Opportunities for SSC among African countries include in the areas of disease prevention and control, production of medical products and essential medicines, harmonization of regulatory processes, and health workforce development among others. However, pitfalls such as poor coordination, inadequate political commitment, lack of conducive policy environments, language barrier and inadequate financing opportunities for SSC initiatives present major dilemma for the use of SSC mechanisms. We conclude that the need for a paradigm shift from vertical to horizontal development cooperation needs no further proof but a call to action. We call on the concerned stakeholders to support the establishment of a systematic approach for use of SSC mechanisms in the health sector of Africa, designation of an African Centre of Excellence for SSC in public health and development of a regional mechanism for monitoring and evaluation of SSC initiatives in Africa.

## Introduction

Implementation of new models of development cooperation has been on the increase lately [1]. Coupled with this are calls for increased use of horizontal development cooperation mechanisms such as South-South Cooperation (SSC) as a mean to enhance aid effectiveness in the health sector of developing countries [2–4]. The calls are fuelled by the often unfavourable terms of North-South Cooperation (NSC), [5, 6] amplified by the need for self-determination, solidarity, sustainable home-grown development and more aid effectiveness among countries of the global south. SSC is also seen as a tool for ensuring equity between developed and developing countries and an opportunity to overcome colonial aid legacy [7, 8]. It is believed that SSC strengthens the principles of the Paris Declaration and Accra Agenda for Action both of which stresses the need for aid effectiveness as well as the Busan Agreement and the Sustainable Development Goals (SDGs) which calls for more inclusive partnerships in development [9, 10]. Historically, SSC referred to the process of exchanging knowledge and resources among countries of the global south. The High-level UN conference on SSC (2009) provides a more comprehensive operational definition of SSC which is *'a process whereby two or more developing countries pursue their individual and/or shared national capacity development objectives through exchanges of knowledge, skills, resources and technical know-how and through regional and interregional collective actions, including partnerships involving Governments, regional organizations, civil society, academia and the private sector, for their individual and/or mutual benefit within and across regions'* [11]. Other form of development cooperation is the Triangular Cooperation (TC), a south-south partnership, led by two or more developing countries but supported by a developed country, multilateral organization or international foundation [11].

Recent public health events in Africa such as the recurrent outbreaks of endemic and emerging infectious diseases like Ebola, Cholera, Yellow Fever, Zika, Dengue and Rift Valley fever [12–15] in areas which hitherto never experienced such have put to test, the capacity and knowledge to respond in the face of increasing incidence of non-communicable diseases [16] and weak health systems. As such a call for a paradigm shift in development cooperation cannot be more apt at this time [17]. Furthermore, the domestication of the SDGs, with the renewed opportunity to address the health system and public health challenges further buttresses the need for more effective development cooperation among countries of the south. While NSC remains the flagship for health development aid in Africa, it is often fraught with inappropriate technology, lack of understanding of the developing countries context, and lack of equality in partnership thus calling for increasing horizontal partnerships among countries of the global south [18]. The foregoing underscores the need for better collaboration, experience sharing and capacity building among African countries given the similarities in their health and development contexts. In this case series paper, based on World Health Organization (WHO) [19] principle that cooperation among countries can be an effective tool to strengthen and accelerate health development, share knowledge and experiences to improve health-while also making the most of existing resources and capacities available within countries and across regions; we review recent experiences in application of SSC initiatives to two public health situations in Africa to demonstrate the veracity of this new paradigm. The objectives of the case study are to review the potential areas where SSC could be applied to strengthen health services delivery in Africa, identify the key challenges which may be associated with its implementation and make appropriate recommendations on how to address the challenges. References are made to other forms of international technical cooperation such as TC and Regional Cooperation (RC) but the main thrust of the paper is the use of SSC in health development cooperation.

## Methods

### Case studies: application of SSC, TC and RC to public health situations in Africa

#### Case study 1: the Ebola Virus Disease (EVD) outbreak in West Africa

The 2014/15 Ebola Virus Disease (EVD) outbreak in West Africa infected more than 28,000 persons out of which 11,000 died [20]. At the height of this outbreak, the huge load of cases and their contacts overwhelmed the response capacity of the principally affected countries namely Guinea, Liberia and Sierra Leone [21]. Available local experience, knowledge, logistic and human resources to manage these outbreaks were limited [22–24] while diagnostic and admission capacities were overstretched; resulting in a backlog of suspected and probable cases awaiting confirmatory laboratory tests and admission into Ebola isolation centres. This sustained community transmission of the disease. A number of SSC, RC and TC initiatives were undertaken during this crisis through negotiations between WHO and Regional Economic Communities (RECs) to provide additional capacity to strengthen diagnosis, case management, identification and follow up of contacts, and community mobilization in the affected countries [22]. The African Union mobilized and deployed more than 850 health workers drawn from 18 African countries to Guinea, Liberia and Sierra Leone through its African Union Support for the Ebola Outbreak in West Africa programme [25, 26] while the Economic Communities of West African States deployed additional 150 West African health personnel. These health workers supported case management, infection prevention and control, active surveillance, contact tracing, community mobilization and outbreak coordination [27]. Through these deployments experienced clinicians and nurses from Uganda and Democratic Republic of Congo, countries with long standing experience in EVD management were deployed to support outbreak response in the principally affected countries. Similarly, China [28], South Africa and Nigeria (with support of the European Union) [29] established level 4 EVD diagnostic laboratories in Liberia and Sierra Leone. Cuba also deployed several Brigades of health workers including doctors, nurses, social workers, infection prevention and control specialists and health administrators to support the principally affected countries. These deployments provided the much needed capacity which significantly contributed to the eventual control of the outbreak. Despite the successes, challenges such as language barrier, inadequate funds to deploy the teams and difficulties in registration of the foreign medical workers in the destination countries hampered speedy deployment and effective utilization of the teams. Furthermore, logistic challenges of transporting, housing and equipping large medical teams and lack of readily available and deployable human resources were also experienced [27, 30].

#### Case study 2: health system strengthening experience sharing between Rwanda and Mozambique

In Mozambique, financing of health services delivery is largely dependent on donor funding which peaked in 2010. On the average, donor funding through sector budget support, projects and vertical funds such as United States President's Emergency Plan for AIDS Relief and Global Fund account for more than 70% of the spending on health in the country while government expenditure on health reduced from 13% to 7% between 2006 and 2010. On the other hand, Rwanda has made good progress in the financing of its health sector. The country has developed and is implementing robust health financing policies and strategic plans, enacted health insurance laws and introduced a community-based health insurance (CBHI) scheme which has over 80% national coverage. These boosted sustainable and equitable access to health services, including safety nets for the poor whilst reducing out of pocket expenditure. This enabled the country to improve its health services delivery mechanisms as well as provide bilateral technical support to a number of other African countries in the areas of health system strengthening and financing [2]. To this effect, a Mozambican delegation comprising of officials from the ministries of health, finance and economy, labour and social security undertook a study tour to Rwanda in October 2015 to share experiences and understudy the Rwanda model for financing national health services. During the study tour, the Mozambican delegation was enlightened on various mechanisms and strategies adopted by Rwanda to finance its health sector including tax-based and insurance funding and performance-based funding. The evidence base, pros and cons of each of these financing mechanisms were discussed and participants paid field visits to observe the practical implementation of the performance based funding, CBHI and community health workers scheme. Following the study tour, Mozambique initiated strategic actions such as finalization of health insurance schemes for public servants and the military, advocacy for health insurance subsidies to make the system sustainable and development of a proposal for pilot testing of CBHI in the country. While the study tour experienced challenges such as inadequate funding, long period of negotiations between the two countries and language barrier which are similar to those of the Ebola deployments, this did not reduce the potential benefits derivable from the exercise.

## Discussion

Africa is at a crossroad; more than ever, the challenge of poor human development, inadequate financial and logistics resources, climate change, and recurrent outbreaks of emerging infectious diseases and natural disasters continues to retard socioeconomic growth and negatively impact on its health systems. The similarities in the socioeconomic, cultural and public health contexts of African countries are opportunities for home-grown efforts to improve development cooperation among them. Available literature especially from countries of Latin America and the Caribbean and the two case studies presented in this paper provide evidence to support scaling up of the use of horizontal development coordination mechanisms to strengthen public health services delivery and socioeconomic development among African countries [31]. The debate is therefore not whether to use the SSC mechanisms for public health services strengthening in Africa or not but how to effectively use it [17]. African countries could reap immense benefits such as cost savings through application of economy of scale strategies in the joint production or procurement of medical products, better bargaining power through joint negotiations, synergy in cross border collaboration and coordination of public health activities such as immunization campaigns, outbreak and humanitarian response. Other benefits include continuity in implementation of health programme across sub-regions through the use of regional commitments which are binding on participating countries, ownership and direct impact of actions on beneficiaries [31]. The use of SSC for health could also be an opportunity to strengthen regional integration. Our case studies illustrate the different dimensions to which SSC mechanisms can be applied to improve public health services delivery and also highlight some of the key challenges which would need to be addressed to ensure its effective use. Clear opportunities for SSC among African countries could be demonstrated in the areas of diseases prevention and control, [32] production of essential medicines, medical products [33], vaccines and harmonization of regulatory processes [34], institutional capacity building and health workforce development [35], direct delivery of specialized health services, public health experience sharing, cross border collaboration and coordination of services delivery and implementation of the international health regulations among others.

Pitfalls such as poor coordination, inadequate political commitment, lack of conducive policy environments, language barrier and inadequate financing opportunities for SSC initiatives have been highlighted as challenges, observed in both case studies, and present a major dilemma for the use of SSC mechanisms in Africa. Other challenges such as lack of evidence from implementation of SSC in other regions [7], lack of information required for effective monitoring and evaluation of outcomes and impact of SSC initiatives and a regional oversight mechanism for issues such as licensing of health workers, regulation of essential medicines and medical products all needs to be addressed in order to benefit from the vast opportunities offered by SSC. The effective and systematic use of SSC mechanisms to strengthen health systems and public health services delivery in Africa would thus require: 1) assessment and identification of common public health problems and objectives in the region to which SSC could be applied; 2) mapping of available financial and human resources which could be harnessed to support the implementation of SSC initiatives across the continent; 3) establishment of a fund for SSC similar to the African Public Health Emergency Fund (APHEF) in the regional banks such as the African Development Bank. Incorporation of SSC mechanisms into existing funds such as APHEF could also be an opportunity to address the funding challenges associated with SSC implementation. Furthermore, increased advocacy to African countries to include funds for SSC in their health budgets would also be a valuable option; 4) development of appropriate regional public health SSC strategies and policy documents which outlines how challenges such as oversight and regulations should be addressed and advocacy for the inclusion of SSC in existing regional and countries policies would also be critical; 5) establishment of regional and national institutional mechanisms for coordination, monitoring and evaluation of SSC/TC activities; 6) overcoming cultural and linguistic barriers to ensure optimum cooperation among countries especially those with similar historic and cultural roots; and to provide opportunities for expansion of SSC activities beyond regional, cultural and linguistic borders would also be needed [17].

Establishing a framework for effective implementation of the foregoing requires involvement of multiple stakeholders. The RECs, governments, private sector, the academia and the UN needs to be fully engaged to provide the overall platform for establishing SSC mechanisms and to advocate to their member states and other stakeholders to strengthen policy dialogue and resource mobilization for SSC in health. Public-private partnerships are crucial to bring the manufacturing sector on board in the areas of production and resource mobilization. National and regional public health organizations could provide the required technical and financial assistance to establish regional and national platforms for public health SSC. Furthermore, they could provide opportunities to link countries in need with appropriate support, provide technical expertise to manage and mobilize resources for public health SSC initiatives [36]. Importantly, African countries as the main duty bearers of their health development agenda should provide the overall leadership for implementation of public health SSC initiatives. The National Public Health Institutes which are proposed as part of the establishment of the Africa Centres for Disease Control [37], and the recently launched SDG Africa Centre [38] could provide a platform for developing capacity for and coordinating the increasing use of SSC mechanisms for public health services delivery on the continent. The community who are the ultimate beneficiaries of SSC initiatives, the civil society and local organizations who are present at the grassroots and can support community-based health initiatives also have critical roles to play.

## Conclusion

Available literature and lessons from the two case studies presented in this paper show that SSC is an opportunity to strengthen national ownership, self-reliance and to harness existing capacities to address common and peculiar African public health problems including achievement of the post-2015 health development agenda and SDG targets. The foregoing demonstrates that the need for a paradigm shift from vertical to horizontal development cooperation needs no further proof but a call to action. We therefore call on concerned stakeholders to support establishment of systems which could facilitate organized use of SSC mechanisms to strengthen public health services delivery in Africa. Such a system should include a regional platform, policy guidelines and institutional arrangements for its implementation. Specifically, regional public health SSC policies and strategies should be developed and implemented. Second, we call for designation of an African Centre of Excellence for international public health cooperation within an existing public health institution on the continent. The Centre should be tasked with conducting in-depth analysis of the enabling factors required for successful application of SSC to public health in Africa and to support coordination, research, knowledge sharing and establishment of a community of practice for SSC in public health. Lastly, we advocate for development of a regional mechanism for supervision, monitoring and evaluation of SSC and TC initiatives.

## Competing interests

The authors declare there exists no competing interests.
